# Neonatal gut colonization by *Bifidobacterium* is associated with higher childhood cytokine responses

**DOI:** 10.1080/19490976.2020.1847628

**Published:** 2020-12-04

**Authors:** Hardis Rabe, Anna-Carin Lundell, Fei Sjöberg, Annika Ljung, Anna Strömbeck, Monica Gio-Batta, Cristina Maglio, Inger Nordström, Kerstin Andersson, Intawat Nookaew, Agnes E. Wold, Ingegerd Adlerberth, Anna Rudin

**Affiliations:** a Institute of Biomedicine, Department of Infectious Diseases, The Sahlgrenska Academy at the University of Gothenburg, Gothenburg, Sweden; b Institute of Medicine, Department of Rheumatology and Inflammation Research, The Sahlgrenska Academy at the University of Gothenburg, Gothenburg, Sweden; c Institute of Biomedicine, Department of Microbiology and Immunology, The Sahlgrenska Academy at the University of Gothenburg, Gothenburg, Sweden; d Wallenberg Centre for Molecular and Translational Medicine, University of Gothenburg, Gothenburg, Sweden; e Department of Biomedical Informatics, College of Medicine, University of Arkansas for Medical Sciences, Little Rock, AR, USA

**Keywords:** Gut microbiota, cytokine responses, T cell activation, next generation sequencing, children

## Abstract

The gut microbiota is a major stimulus for the immune system, and late acquisition of bacteria and/or reduced complexity of the gut flora may delay adaptive immune maturation. However, it is unknown how the gut bacterial colonization pattern in human infants is related to T cell activation during early childhood. We followed 65 Swedish children in the FARMFLORA cohort, from birth up to 3 years of age. In fecal samples collected at several time points during the first year of life, the gut colonization pattern was investigated with the use of both 16S rRNA next generation sequencing (NGS) and culture-based techniques. This was related to production of IL-13, IL-5, IL-6, TNF, IL-1β and IFN-γ by PHA-stimulated fresh mononuclear cells and to proportions of CD4^+^ T cells that expressed CD45RO at 36 months of age. Both NGS and culture-based techniques showed that colonization by *Bifidobacterium* at 1 week of age associated with higher production of IL-5, IL-6, IL-13, TNF and IL-1β at 36 months of age. By contrast, gut colonization by *Enterococcus, Staphylococcus aureus* or *Clostridium* in early infancy related inversely to induced IL-13, IL-5 and TNF at 3 years of age. Infants with elder siblings produced more cytokines and had a larger fraction of CD45RO^+^ T cells compared to single children. However, controlling for these factors did not abolish the effect of colonization by *Bifidobacterium* on immune maturation. Thus, gut colonization in early infancy affects T cell maturation and *Bifidobacterium* may be especially prone to induce infantile immune maturation.

## Introduction

The gut microbiota is a major stimulus for the immune system. Hence, late acquisition of bacteria and/or reduced complexity of the gut flora may delay adaptive immune maturation. Lamina propria lymphocytes from germ-free mice are fewer than those from mice with a normal gut flora and these lymphocytes also produce less of both the Th1-related cytokine IFN-γ and the Th2-related cytokines IL-4 and IL-13.^1^ In humans, the influence of the infantile gut colonization pattern on the adaptive immune cell development is still under early investigation. We have previously shown that early colonization by *Bifidobacterium* spp or *Escherichia coli* (*E. coli*) is associated with higher number of circulating CD27^+^ memory B cells at 4 and 18 months of age.^2^ However, if the activated CD4^+^ T cell population and cytokine responses during the first years of life are associated with the gut bacterial colonization pattern during infancy is still not known.

The establishment of the gut microbiota starts during or directly after birth, when the neonate is first exposed to bacteria. Facultative anaerobic bacteria, including *E. coli* and *Enterococcus* spp, are early colonizers of the infantile gut, which are followed by obligate anaerobes such as *Bifidobacterium, Bacteroides* and *Clostridium*.^3,4^ Successively, more strictly anaerobic bacteria colonize the gut until a complex microbiota dominated by anaerobes is fully established at 1–3 years of age.^3–6^ However, the infantile colonization pattern has changed over the last decades,^7–11^ probably reflecting decreased exposure to fecal bacteria in hygienic societies. Thus, colonization by *E. coli* and *Bacteroides* occurs later today than before,^7–11^ while classical skin bacteria, such as coagulase-negative staphylococci and *S. aureus*, are now common members of the infantile gut microbiota.^7,12^ The anaerobic bacterium *Clostridium difficile*, which expands in microbiota of low complexity, has also become more common.^13^

In the FARMFLORA birth-cohort, we have prospectively followed 65 Swedish children from birth up to three years of age. With the use of both next generation sequencing and traditional bacterial culture, we examined whether gut colonization by various bacteria during the first six months of life was associated with PHA-induced cytokine production by fresh mononuclear cells at 36 months of age.

## Material and methods

### Subjects and collection of blood, rectal and fecal samples

Blood, rectal and fecal samples were obtained from participants in the prospective FARMFLORA study, which includes 65 children (33 boys and 32 girls) born at term (≥38 gestational weeks) in rural areas of Southwest Sweden.^2,14,15^ Twenty-eight of the children lived on small dairy farms, while 37 lived in the countryside, but not on a farm. Peripheral blood samples were obtained at 36 months of age (n = 50). All blood samples were collected in heparin tubes. Rectal swabs were obtained at 3 days of age and fecal samples at 1 and 2 weeks, and at 1, 2, 4, 6 and 12 months of age. Informed consent was obtained from the parents, and the study was approved by the Human Research Ethics Committee of the Medical Faculty, University of Gothenburg, Sweden (Dnr 363–05).

### Flow cytometry

The proportions of CD4^+^ T cells that were CD45RO-positive were analyzed by flow cytometry within 72 hours after sampling, as previously reported in detail.^14–16^ The following antibodies were used: PerCP-conjugated anti-CD4 (clone SK3, BD Bioscience) and PE-conjugated anti-CD45RO (clone UCHL-1, BD Bioscience). Isotype controls were purchased from BD Bioscience. A FACSCalibur (BD Bioscience) equipped with CellQuestPro software was used to examine stained samples and flow cytometry data were analyzed using Flow Jo software (TreeStar, Ashland Oregon).

### Cell culture and cytokine determinations

Blood mononuclear cells sampled at 36 months of age were analyzed for cytokine production after PHA stimulation as reported in detail previously.^15^ Concentrations of IL-1β, IL-6, TNF, IFN-γ, IL-5 and IL-13 in the supernatants were determined using Flow Cytomix (eBioscience, Vienna, Austria) followed by flow cytometry on a FACSCanto II (BD Biosciences) equipped with a FACSDiva software.

### Culturing and identification of living gut bacteria

Rectal samples were cultured for presence of major groups of facultative gut bacteria (yes/no) and fecal samples were diluted and cultured quantitatively for major groups of facultative and anaerobic gut bacteria, as previously described.^7^ **Supplemental Table 1** depicts culture conditions and methods used to identify living bacterial species or groups of bacteria. All bacteria detected by culture, but not NGS, are analyzed at genus (*Bifidobacterium, Bacteroides, Lactobacillus, Enterobacterium non-E.coli, Enterococcus, Clostridium*) or at species level (*Staphylococcus aureus, Escherichia coli* and *Clostridium difficile*) and denoted with a suffix throughout the paper. To evaluate the anaerobic/aerobic ratio, bacterial counts of facultative bacteria were determined from aerobic growth on Colombia blood agar. Total anaerobic counts were determined from anaerobic growth on Brucella blood agar, after subtracting the counts of isolates also growing aerobically. The ratio between anaerobic/aerobic bacteria was then calculated by dividing the counts of anaerobic bacteria by the counts of facultative bacteria for each child.

### 16S rRNA next generation gene sequencing analysis

Next generation sequencing of 16S rRNA genus was applied on fecal samples collected at 1 week, 1 month and 6 months of age. Bacterial DNA was extracted from 180 mg feces with the use of QIAamp DNA stool mini kit (QIAGEN AB, Sollentuna Sweden), and an extra purity step was added to increase the DNA yield as described previously.^17^ In short, four glass beads (3 mm diameter) and 0.5 g zirconia beads (0.1 mm diameter) were added to ASL buffer. The fecal samples were homogenized (2x40 sec at 6 m/sec) with the use of Fastprep FP120 cell disrupter (Thermo Savant, Illkirch France), incubated at 95° C for 5 min and shaken for 30 min at 4° C with use of an IKA vibrax VXR shaker (IKA-Werke GmbH, Staufen, Germany).

DNA amplification occurred in two PCR reaction steps. For the first PCR amplification the V3-V4 region of the 16S rRNA gene was amplified using the following primers, 34IF (5´-TCGTCGGCAGCGTCAGATGTGTATAAGAGACAGCCTACGGGNGGCWGCAG-3´) and 785 R (5´-GTCTCGTGGGCTCGGAGATGTGTATAAGAGACAGGACTACHVGGGTATCTAATCC-3´).^18^ Detail on reaction volumes and PCR amplification conditions for both PCR amplification steps are shown in **Supplemental Table 2**. DNA products from each PCR reaction were purified using AmPure XP magnetic beads (Beckman Coulter, USA). DNA from the first PCR was quantified using a Qubit dsDNA high sensitivity assay kit (Thermo Scientific, USA). In the second amplification PCR single multiplexing was performed with the use of an 8-bp index that was added to both the forward and reverse primers (described in **Supplemental Table 3**). Amplification length of the second PCR was approximately 600 bp and determined with the use of Agilent 2200 Tapestation and the DNA screen Tape analysis kit (Agilent Technologies Sweden AB Kista, Sweden). Lastly, purified PCR product was diluted to a concentration of 4 ng/µl, pooled into equal amounts and sent for sequencing to Science for Life Laboratory (Stockholm, Sweden).

The 16S rRNA gene amplifications were sequenced on an Illumina MiSeq system, with a 2 × 300 sequencing setup (Illumina Corp. San Diego, CA, USA). bcl2fastq_v2.19.1.403. (CASAVA software suite) was used to convert Bcl to FastQ. The sequenced data were quality checked with the FastQC ver.0.11.5 software and processed with QIIME 2.^19^ The quality of the primed reads was assessed by QIIME 2 and the sequence trimming, and truncation base position values were determined. Then DADA2 plugin was used to obtain the amplicon sequencing variants (ASVs) of individual sample. The merged ASVs were then clustered into 97% OTUs (operational taxonomic units) through vsearch plugin^20^ with the default parameter to reduce complexity of the data for further taxonomic assignment. The representative OTU sequences were mapped and assigned taxonomy based on SILVA (v132) SSU rRNA reference sequenced database. Reads per sample, ASV per sample and OTUs per sample for each child and time point are depicted in **Supplemental Table 4**.

### Statistical analysis

Multivariate factor analysis was used to assess the relationship between cytokine responses at 36 months of age and the early gut bacterial colonization pattern, environmental factors or the percentage of memory T cells. The read count of all NGS samples was firstly normalized to have the similar library size using DESeq2^21^ and used for further analysis as abundance levels. Variables that were not normally distributed were log transformed in multivariate factor analysis. All multivariate factor analyses were performed with the use of SIMCA-P+ software (version 15, Sartorius Stedem Biotech, Umeå, Sweden) and each model performed are described in Table 1. Principal component analysis (PCA) was used to obtain an overview of groupings and trends regarding T cell activation and the environmental factors. Orthogonal projection to latent structures by means of partial least squares (OPLS) was implemented to correlate the X and Y data matrices, where X represented gut colonization by the different bacteria and Y represented the levels of PHA-induced cytokines or the proportions of CD45RO^+^CD4^+^ T cells. The final OPLS loadings column plots are based on X–variables with variable influence of projection values (VIP-values) >1.5 for NGS and >1.2 for culture technique (Table 1). VIP-values are used to identify unimportant predictors for the overall model that can be eliminated. The quality of the multivariate models was assessed based on the parameters R2 and Q2, i.e. the percentages of the variation of the data set explained (R2) and predicted (Q2) by the model, respectively.^22^ Cross-validation is used to determine R2 and Q2, in which SIMCA by default generates several alternative models that are based on excluding data groups from the original data set and then calculating the differences between these models and the original model.^22^ Univariate analyses were exclusively performed for Y and X–variables that showed the strongest associations in the respective OPLS models, i.e. variables that displayed large bars with small error bars. Univariate analyses were performed by Mann-Whitney U test or Spearman’s rank correlation test (GraphPad Prism, GraphPad, San Diego, USA) as described in the figure legends. To study the independent association between bacterial colonization, environmental factors and cytokine responses, multiple linear regression analysis was performed with SPSS (IBM Corporation, New York, NY). Only variables that were significant in univariate analysis were included in the linear regressions. For all statistical analyses, *P* ≤ 0.05 was considered as significant (* *P* ≤ 0.05, ** *P* ≤ 0.01 and *** *P* ≤ 0.001).

**Figure 1.**
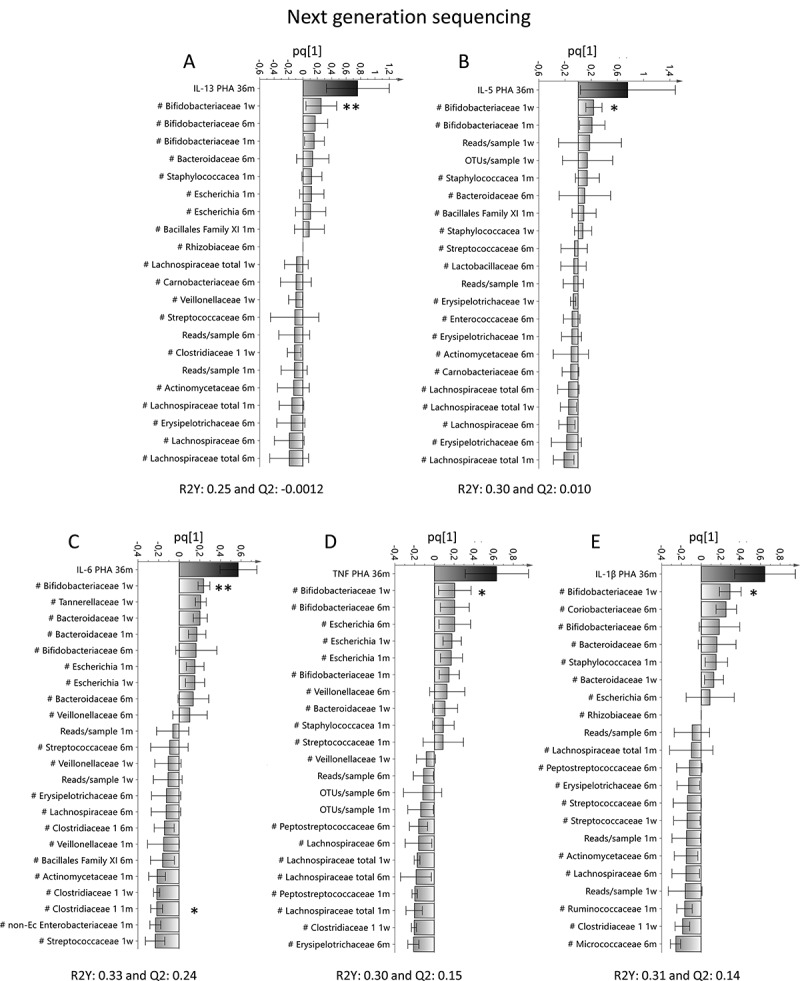
Associations between T cell activation and the infantile gut microbiota determined by 16S rRNA NGS. Orthogonal projection to latent structures by means of partial least squares (OPLS) column plots depicting the association between early bacterial colonization (X–variables) analyzed by 16S rRNA NGS and the PHA-induced response of IL-13 (a), IL-5 (b), IL-6 (c), TNF (d) or IL-1β (e) from mononuclear cells at 36 months of age. X–variables that lie in the same direction as the cytokine response are positively associated, whereas parameters on the opposite direction are inversely related to concentrations of IL-13, IL-5, IL-6, TNF or IL-1β. Each column displays an uncertainty bar with 95% confidence interval. Asterix depicts associations found to be statistical significant by the use of Mann Whitney’s U test (*p < .05 and **p < .01)

**Figure 2.**
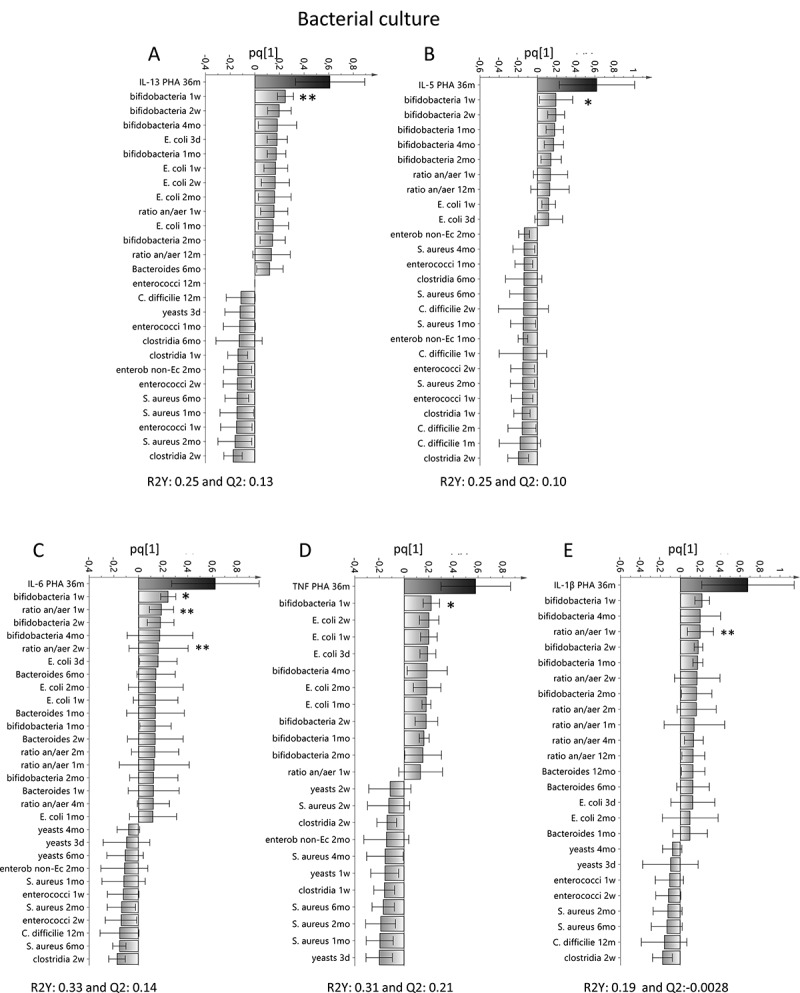
Associations between T cell activation and the infantile gut microflora determined by bacterial culture. OPLS plots depicting the association between early bacterial colonization (X–variables) analyzed by culture and the PHA-induced response of IL-13 (a), IL-5 (b), IL-6 (c), TNF (d) or IL-1β (e) from mononuclear cells at 36 months of age. X–variables that lie in the same direction as the cytokine response are positively associated, whereas parameters on the opposite direction are inversely related to concentrations of IL-13, IL-5, IL-6, TNF or IL-1β. Each column displays an uncertainty bar with 95% confidence interval. Asterix depicts associations found to be statistical significant by the use of Mann Whitney’s U test or Spearman rank correlation test (*p < .05 and **p < .01)

**Figure 4.**
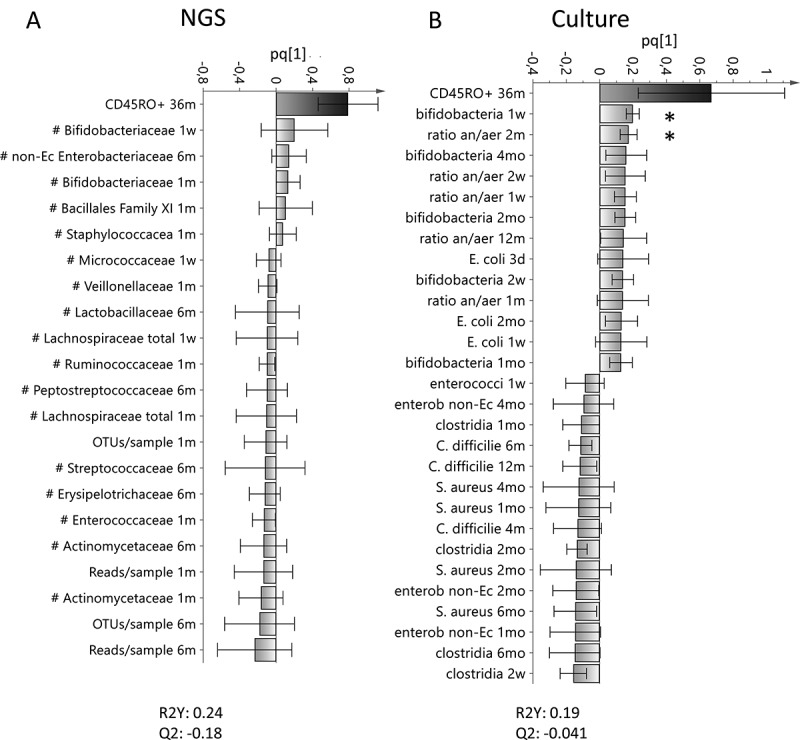
Associations between the proportion of CD45RO^+^CD4^+^ memory T cells and the infantile gut microflora. OPLS column plots depicting the association between early bacterial colonization (X–variables) analyzed with 16S rRNA NGS (a) or culture (b) and the proportion of activated CD4^+^ that express CD45RO at 36 months of age. X–variables that lie in the same direction as the proportion of activated CD4^+^ that express CD45RO are positively associated, whereas parameters on the opposite direction are inversely related. Each column displays an uncertainty bar with 95% confidence interval. Asterix depicts associations found to be statistical significant by the use of Mann Whitney’s U test or Spearman rank correlation test (*p < .05)

**Figure 5.**
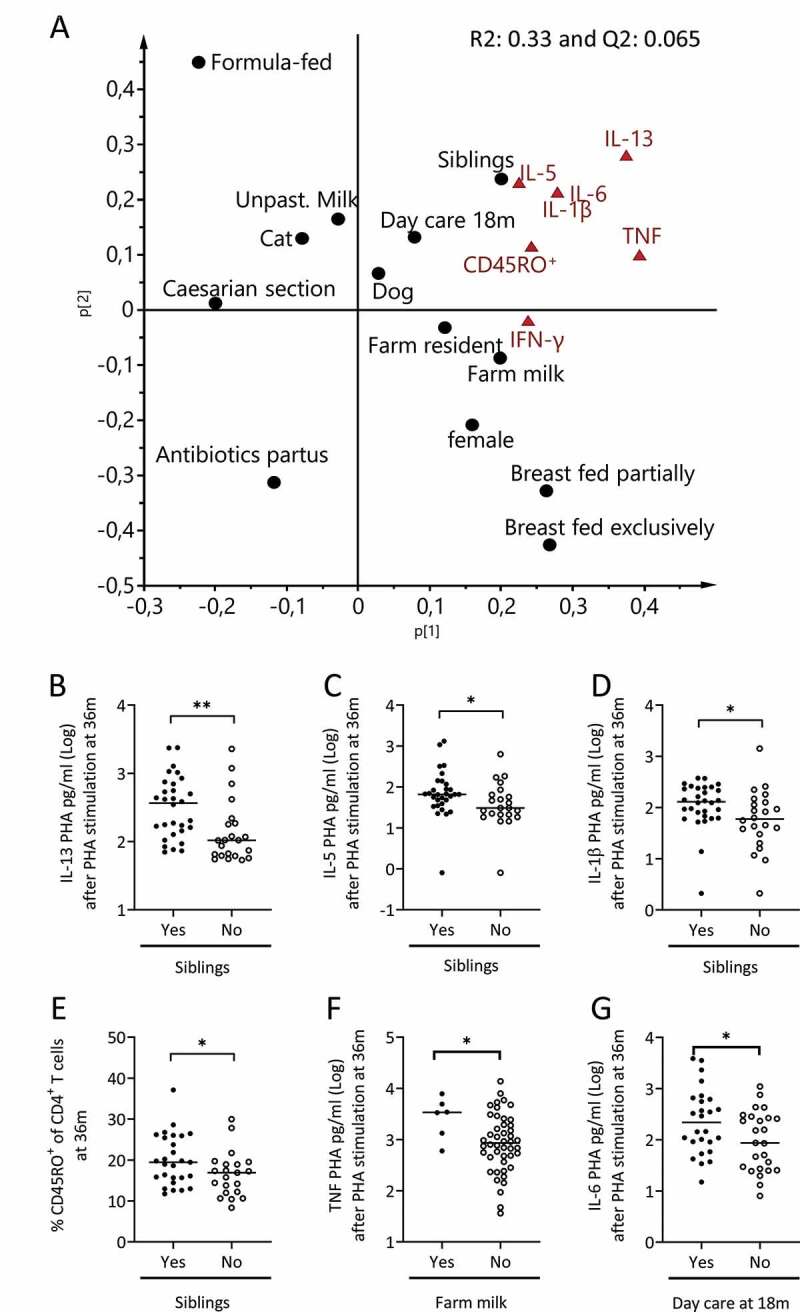
The relation between environmental factors and the capacity of mononuclear cells to produce cytokines. (a) PCA loading scatter plot depicting the associations between the production of cytokines in response to PHA-stimulation by mononuclear cells obtained at 36 months of age and environmental factors during infancy. Parameters projected on the same side of the Y-axis are positively associated, whereas parameters projected on opposite sides of the Y-axis are inversely related to each other. (b-e) The production of IL-13 (b), IL-5 (c), IL-1β (d) by mononuclear cells and the percentage of CD45RO^+^ cells within the CD4^+^ T cell population (e) at 36 months of age by children who either had elder siblings or not. (f-g) the concentration of TNF (f) or IL-6 (g) after PHA-stimulation of mononuclear cells at 36 months of age by children drinking farm milk or not (F) or attending day care at 18 months of age (G). Each dot represents one child, and the horizontal bar represents the median value. Mann Whitney’s U test, *p < .05 and **p < .001

**Table 1.** Multivariate factor analyses

Figure	Analysis	Y-variable	X–variables	VIP-value
Figure 1a	OPLS^a^	IL-13	NGS detected bacteria^d^	>1.5
Figure 1b	OPLS^a^	IL-5	NGS detected bacteria^d^	>1.5
Figure 1c	OPLS^a^	IL-6	NGS detected bacteria^d^	>1.5
Figure 1d	OPLS^a^	TNF	NGS detected bacteria^d^	>1.5
Figure 1e	OPLS^a^	IL-1β	NGS detected bacteria^d^	>1.5
Figure 2a	OPLS^a^	IL-13	Culture detected bacteria^e^	>1.2
Figure 2b	OPLS^a^	IL-5	Culture detected bacteria^e^	>1.2
Figure 2c	OPLS^a^	IL-6	Culture detected bacteria^e^	>1.2
Figure 2d	OPLS^a^	TNF	Culture detected bacteria^e^	>1.2
Figure 2e	OPLS^a^	IL-1β	Culture detected bacteria^e^	>1.2
Figure 4a	OPLS^a^	CD45RO	NGS detected bacteria^d^	>1.5
Figure 4b	OPLS^a^	CD45RO	Culture detected bacteria^e^	>1.2
Figure 5a	PCA^b^		Cytokine responses^c^ Environmental factors^f^	
Supplemental Figure 1a	OPLS^a^	CD45RO	Cytokine responses^c^	
Supplemental Figure 5a	OPLS^a^	IFN-γ	NGS detected bacteria^d^	>1.5
Supplemental Figure 5b	OPLS^a^	IFN-γ	Culture detected bacteria^e^	>1.2
Supplemental Figure 6A	OPLS^a^	Ceasaran section	Cytokine responses^c^ CD45RO	
Supplemental Figure 6B	OPLS^a^	Female	Cytokine responses^c^ CD45RO	
Supplemental Figure 6C	OPLS^a^	Antibiotics delivery	Cytokine responses^c^ CD45RO	
Supplemental Figure 6D	OPLS^a^	Formula fed	Cytokine responses^c^ CD45RO	
Supplemental Figure 6E	OPLS^a^	Breast fed exclusively	Cytokine responses^c^ CD45RO	
Supplemental Figure 6F	OPLS^a^	Breast fed partially	Cytokine responses^c^ CD45RO	
Supplemental Figure 6G	OPLS^a^	Farm	Cytokine responses^c^ CD45RO	
Supplemental Figure 6H	OPLS^a^	Un-pasteurized milk	Cytokine responses^c^ CD45RO	
Supplemental Figure 6I	OPLS^a^	Cat	Cytokine responses^c^ CD45RO	
Supplemental Figure 6J	OPLS^a^	Dog	Cytokine responses^c^ CD45RO	

^a^OPLS = Orthogonal projection to latent structures by means of partial least squares

^b^PCA = Principal Component Analysis

^c^Cytokine responses include: IL-13, IL-5, IL-6, IL-1β, TNF and IFN-γ

^d^Bacteria detected by NGS that include Actinomycetaceae, Bifidobacteriaceae, Corynebacteriaceae, Micrococcaceae, Propionacteriaceae, Atopobiaceae, Coriobacteriaceae, Eggerthellaceae, Bacteroidaceae, Barnesiellaceae, Prevotellaceae, Rikenellaceae, Tannerellaceae, Bacillales Family XI, Staphylococcacea, Carnobacteriaceae, Enterococcaceae, Lactobacillaceae, Streptococcaceae, Clostridiaceae, Family XI, Family XIII, Lachnospiraceae, Peptostreptococcaceae, Ruminococcaceae, Erysipelotrichaceae, Acidaminococcaceae, Veillonellaceae, Desulfovibrionaceae, Burkholderiaceae, Neisseriaceae, Akkermansiaceae, non-E. coli Enterobacteriaceae, Escherichia, and Pasteurellaceae

^e^Bacteria detected by culture techniques that include *Enterococcus* spp, enterobacterium non- *Ecoli, Clostridium spp, Clostridium difficilie, Lactobacillus spp, Staphylococcus aureus, E.coli, Bifidobacterium spp*. Yeast and ratio of anaerobic/aerobic data are also included in these analysis.

^f^Environmental factors that include being born by Cesarian section, sex, antibiotics at delivery (mother), having older siblings, children who were breast fed or formula fed partially or exclusively, living on a farm, intake of un-pasteurized milk or farm milk and having cats or dogs.

## Results

### Cytokine responses correlate with the proportion of activated T cells

The fraction of circulating CD45RO^+^CD4^+^ T cells increases gradually during infancy as does production of several cytokines after stimulation with the T cell mitogen PHA.^14,15^ We related cytokine pattern after PHA stimulation and the fraction of CD45RO^+^CD4^+^ T cells and found that PHA-induced IL-5 and IL-13 production was significantly associated with the proportions of activated CD45RO^+^CD4^+^ T cells at 36 months of age (**Supplemental** Figure 1a-**C**). TNF, IL-1β, IFN-γ or IL-6 responses were unrelated to CD45RO^+^CD4^+^ T cells (univariate analysis not shown). Thus, children with higher proportions of activated circulating CD4^+^ T cells also have mononuclear cells with higher capacity to produce IL-13 and IL-5.

### *Neonatal colonization by* Bifidobacterium *is associated with higher capacity to produce cytokines later in childhood*

To study how the cytokine responses and the activated CD4^+^ T cell proportion during the first years of life were associated with the gut bacterial colonization pattern during infancy, we performed OPLS analysis that included cytokine levels (Y-variables) and bacteria detected by either NGS or culture techniques (X–variables). All bacteria detected by NGS were analyzed at family level, whereas bacteria detected by culture techniques were analyzed at genus or species level. The relative mean abundance of bacteria at family level at 1 week, 4 and 6 months of age is shown in **Supplemental Figure 2**. We found that higher levels of IL-13, IL-5, IL-6, TNF and IL-1β were most strongly associated with higher abundance of Bifidobacteriaceae at 1 week of life (Figure 1; NGS) and presence of *Bifidobacterium* spp in fecal cultures from the same time point (Figure 2; culture). Higher IL-6 and IL-1β responses were also associated with a high anaerobic/aerobic ratio at 1 and 2 weeks of life (Figure 2c and e; culture). In contrast, IL-13 and IL-5 responses were inversely related to higher abundance of Lachnospiraceae (Figure 1a-b; NGS) and to infantile colonization by *Clostridium* spp, *Enterococcus* spp and *S. aureus* (Figure 2a-b; culture). IL-6, TNF and IL-1β responses were inversely related to higher abundance of Streptococcaceae and Clostridiaceae (Figure 1c-e; NGS) and to infantile colonization by *Clostridium* spp, *Enterococcus* spp and *S. aureus* (Figure 2c-e; culture).

**Figure 3.**
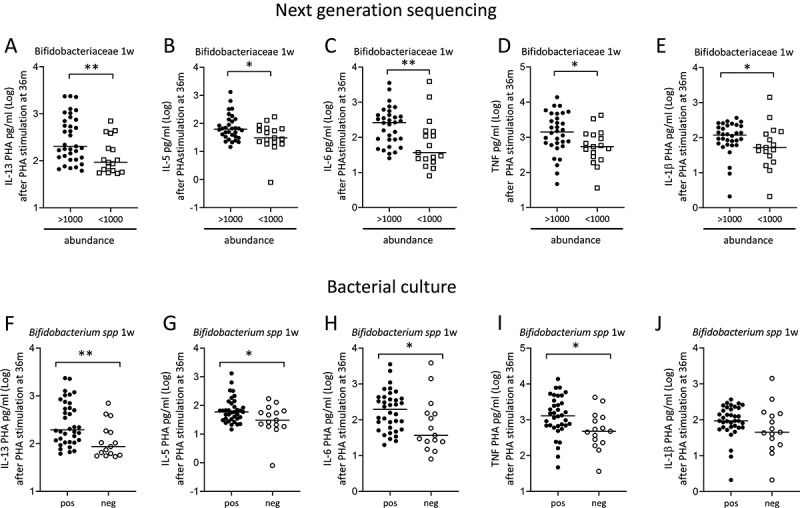
Children colonized with high abundance of Bifidobacteriaceae or colonized by *Bifidobacterium* spp in early infancy have mononuclear cells with higher capacity to produce cytokines after PHA-stimulation. The concentration of IL-13 (a), IL-5 (b), IL-6 (c), TNF (d) and IL-1β (e) after PHA-stimulation of mononuclear cells from children at 36 months of age who had an abundance above or below 1000 Bifidobacteriaceae in feces at 1 week of life. The concentration of IL-13 (f), IL-5 (g), IL-6 (h), TNF (i) and IL-1β (j) after PHA-stimulation of mononuclear cells from children at 36 months of age who were colonized or not by *Bifidobacterium* spp at 1 week of life. Each dot represents one child, and the horizontal bar represents the median value. Mann Whitney’s U test, *p < .005 and **p < .001

In univariate analysis, the cut-off level for high and low Bifidobacteriaceae, i.e. abundance of 1000, was based on the finding that the abundance of these bacteria at 1 week of age formed two separate groups when correlated to cytokines responses (**Supplemental Figure 3A-E)**. Univariate analysis corroborated that children with higher abundance of Bifidobacteriaceae at 1 week of age had mononuclear cells with a higher capacity to produce IL-13, IL-5, IL-6, TNF and IL-1β at 36 months of age compared to children with lower abundance of these bacteria (Figure 3a-e; NGS). Similarly, colonization detected by culture showed that children colonized by *Bifidobacterium* spp at 1 week of age had higher IL-13, IL-5, IL-6 and TNF, but not IL-1β, responses at 36 months of age (figure 3f-j; culture). Children with higher ratio of anaerobic/aerobic bacteria in the feces at 1 and 2 weeks of age had significantly higher IL-6 responses (*r* = 0.45 *p* = .002, and *r* = 0.43 *p* = .003, respectively). Higher ratio of anaerobic/aerobic bacteria at 1 week of life also correlated significantly with higher IL-1β responses at 36 months of life (*r* = 0.44 *p* = .003). Univariate analysis showed that high abundance of Clostridiaceae at 1 month related to lower capacity to produce IL-6 at 36 months of age (**Supplemental Figure 4A-B**; NGS). Moreover, children colonized by *Enterococcus* spp at 1 week produced lower IL-13 and IL-5 levels at 36 months of age compared to cells from non-colonized children (**Supplemental Figure 4 C-D**; culture), and children colonized by *Clostridium* spp at 2 weeks or *S. aureus* at 1 month of life produced lower levels of IL-6 and TNF compared to children who were not colonized by these bacteria (**Supplemental Figure 4E-F**; culture).

Multivariate factor analysis also showed a positive association between higher proportions of CD45RO^+^CD4^+^ T cells at 36 months of age and higher abundance of Bifidobacteriaceae, colonization by *Bifidobacterium* spp at 1 week of age, and a higher ratio of anaerobic/aerobic bacteria at 2 months of age (Figure 4a and b, respectively). Accordingly, univariate analysis confirmed that children who had higher proportions of circulating CD45RO^+^CD4^+^ T cells at 36 months of age were colonized by *Bifidobacterium* spp at 1 week of age (*p* = .04), and/or had a higher ratio of anaerobic/aerobic bacteria at 2 months of age (*r* = 0.33 *p* = .03). In contrast to the other cytokines assessed, higher IFN-γ responses were associated with higher abundance of Streptococcaceae and Clostridaceae and early colonization by enterobacterium non-*E. coli* and *Clostridium* spp (**Supplemental Figure 5A and B**; NGS and culture respectively). However, none of these associations was confirmed by univariate analysis (data not shown).

Taken together, both NGS and culture techniques showed that children with a gut flora containing *Bifidobacterium* in early infancy or a high ratio of anaerobic/aerobic bacteria had mononuclear cells with higher capacity to produce cytokines later in childhood.

### Relation between environmental factors and T cell activation

Infantile gut bacterial colonization is affected by environmental factors such as delivery mode, feeding habits and the presence of elder siblings.^8,23^ Thus, we studied environmental factors at birth or early infancy that might be associated with cytokine responses at 36 months of life. In PCA analysis, having one or more elder sibling(s), being raised on a farm, drinking farm milk or attending day care at 18 months of age were all projected close to higher capacity to produce cytokines at 36 months of age (Figure 5a). Univariate analysis confirmed that children with elder siblings had higher capacity to produce IL-13, IL-5 and IL-1β as well as a higher percentage of activated CD45RO^+^CD4^+^ T cells at 36 months of age compared to children without elder siblings (Figure 5b-E). Children who consumed some farm milk had mononuclear cells with a higher capacity to produce TNF and IFN-γ (figure 5f and *p* = .0004, respectively) and children who attended day care at 18 months of age had higher IL-6 responses at 36 months of age (Figure 5g). No associations were found between cytokine responses and Cesarean section, sex, antibiotics at delivery (mother), breastfeeding (neither exclusively nor partly), formula intake, being raised on a farm, intake of unpasteurized milk or having pets (**Supplemental Figure 6**).

We next performed multiple regression analyses to examine whether the associations between gut colonization by specific bacteria and enhanced capacity to produce cytokines later in childhood were independent of the presence of environmental factors. In regression analyses only bacteria that were significantly associated with higher capacity of mononuclear cells to produce cytokines were included, i.e. higher abundance of Bifidobacteriaceae or presence of *Bifidobacterium* spp at 1 week of age. Furthermore, only environmental factors that were related to higher cytokine responses were included in the regression analysis, i.e. having siblings, intake of farm milk or attending day care at 18 months of age (Figure 5b-g). Higher abundance of Bifidobacteriaceae or colonization by *Bifidobacterium* spp at 1 week contributed to a higher capacity to produce IL-13, IL-5, IL-6 and TNF at 36 months of age independently of environmental factors (Table 2; NGS and Table 3; culture). Regarding the IL-1β response and proportion of CD45RO^+^CD4^+^ T cells at 36 months of age, neither having elder siblings nor being colonized by *Bifidobacterium* spp at 1 week of age were independently associated with higher responses (Tables 2 and 3). Thus, having siblings at the time of birth, drinking farm milk or attending day care at 18 months of age were not likely to be confounding factors for the associations between early gut colonization by *Bifidobacterium* and an enhanced capacity to produce IL-13, IL-5, IL-6 and TNF by mononuclear cells later in childhood.

**Table 2.** Multiple linear regression analysis regarding the relation between cytokine responses, environmental factors and abundance of Bifidobacteriaceae

	Beta	P value
**A**	**IL-13**^1^	
Elder siblings	0.2	0.2
Bifidobacteriaceae 1w^2^	0.1	**0.009**
**B**	**IL-5**^1^	
Elder siblings	0.2	0.2
Bifidobacteriaceae 1w^2^	0.1	**0.008**
**C**	**IL-1β**^1^	
Elder siblings	0.3	0.08
Bifidobacteriaceae 1w^2^	0.04	0.4
**D**	**IL-6**^1^	
Day care 18 m	−0.2	0.3
Bifidobacteriaceae 1w^2^	0.1	**0.05**
**E**	**TNF**^1^	
Farm Milk	0.1	0.6
Bifidobacteriaceae 1w^2^	0.1	**0.05**

^1^Cytokine levels in supernatants of PHA-stimulated fresh mononuclear cells

from 36 month old children

^2^Abundance levels detected by 16S rRNA NGS

**Table 3.** Multiple linear regression analysis regarding the relation between cytokine responses, environmental factors and colonization by *Bifidobacterium* spp

	Beta	P value
**A**	**IL-13^1^**	
Elder siblings	0.2	0.1
*Bifidobacterium* spp 1w^3^	0.4	**0.008**
**B**	**IL-5**^1^	
Elder siblings	0.2	0.2
*Bifidobacterium* spp 1w^3^	0.3	**0.03**
**C**	**IL-1β**^1^	
Elder siblings	0.3	0.07
*Bifidobacterium* spp 1w^3^	0.2	0.2
**D**	**CD45RO^+^CD4^+^ T cells**^2^	
Elder siblings	3.5	0.06
*Bifidobacterium* 1w^3^	−0.5	0.4
**E**	**IL-6**^1^	
Day care 18 m	−0.3	0.2
*Bifidobacterium* spp 1w^3^	0.5	**0.03**
**F**	**TNF**^1^	
Farm Milk	0.1	0.6
*Bifidobacterium* spp 1w^3^	0.4	**0.03**

^1^Cytokine levels in supernatants of PHA-stimulated fresh mononuclear cells

from 36 month old children

^2^The percentage of CD45RO^+^ cells within the CD4^+^ T cell population at

36 months of age

^3^Bacterial colonization detected by culture techniques

## Discussion

Although animal models have clearly shown the importance of gut bacterial colonization on the development of the immune system,^1,24^ data from humans are sparse. We have reported, using culture-based techniques, that early gut colonization by *Bifidobacterium spp* and *E. coli* correlated positively with the proportion of B cells that express the memory marker CD27 at 4 and 18 months of age.^2^ Here, we show that T cell development and functionality in early infancy may also be shaped by gut colonization. Using either NGS or culture-based techniques, we demonstrate that early colonization by *Bifidobacterium* in infancy is associated with a more mature T cell phenotype at 3 years of age, including T cells with a higher capacity to produce cytokines in response to the mitogen PHA, and a higher proportion of CD4^+^ T cells expressing the memory marker CD45RO. Thus, an early gut microbiota that contains *Bifidobacterium* may enhance T cell activation.

The combination of NGS and culture-based techniques offers several advantages. NGS enables identification of utterly oxygen-sensitive anaerobic bacteria that may not be cultured. Culture-based techniques, on the other hand, have a higher sensitivity for facultative bacteria, which are often present at relatively low population levels and may be missed by NGS.^6^ Further, culture enables identification to deeper taxonomic levels, while DNA-based techniques usually cannot distinguish between different species within a genus and often can only identify the family or even higher taxonomic levels.

In the present study, both NGS and culture-based techniques showed that colonization by *Bifidobacterium* as early as 1 week of life was associated with a stronger mitogen-induced IL-13, IL-5, IL-6 and TNF production and fraction of memory CD45RO^+^CD4^+^ T cells, while IFN-γ levels were unrelated to the early bacterial colonization pattern. However, it has been shown that intestinal colonization by *Bifidobacterium* at 1 month is unrelated to LPS-induced IL-6 and TNF production by mononuclear cells at 12 months of age.^25^ One plausible explanation for this discrepancy could be that we used PHA, which is a T cell mitogen, while LPS activates innate immune cells and in fact de-activates T cells.^26^ In line with our findings, Bangladeshi infants with a high abundance of *Bifidobacterium* in the gut over the first 15 weeks of life display a stronger BCG vaccine-induced memory CD4^+^ T cell response at 2 years of age (BCG vaccination at birth) compared to children with lower abundance of bifidobacteria.^27^ Taken together, these results suggest that an early gut flora containing *Bifidobacterium* is associated with increased adaptive immune activation.

In contrast, a gut flora characterized by more *Enterococcus* spp, *Clostridium* spp and *S. aureus* was associated with lower cytokine responses later in childhood compared to non-colonized children. These bacteria represent bacterial groups that expand in microbiota of low complexity. Facultative bacteria that attain high population levels in the immature microbiota of the newborn infant are suppressed by the expansion of obligate anaerobic bacteria.^12,28^ The same is true for some anaerobes, notably *C. difficile* whose expansion is a sign of a disturbed microbiota.^29^ It is therefore possible that high population rates and counts of *Clostridium* spp, *Enterococcus* spp and/or *S. aureus* in the infantile gut could be a marker of low microbiota diversity, whereas early acquisition of *Bifidobacterium* might be a marker of a more diverse gut flora.^1,8,25,30^

Several environmental and lifestyle factors may influence the gut bacterial colonization pattern, such as delivery mode, diet, antibiotic treatment and the presence of elder siblings,^8,23^ being exposed to antibiotics during delivery and being breastfed.^31^ Indeed, children with elder siblings responded more strongly to PHA stimulation and had a higher proportion of CD45RO expressing CD4 T cells, but these associations were attenuated when controlling for colonization by *Bifidobacterium*. In addition, some other exposure, such as drinking farm milk or attending daycare at 18 months of age was also related to increased production of certain cytokines, but were not independent factors in multiple regression analysis when including gut bacteria.

One major strength of this study is the use of both NGS and culture techniques to study the gut colonization as it allowed us to identify both bacteria that are difficult to culture as well as live facultative bacteria. Another major strength is the prospective nature of the study as the immunological effects were followed up to three years after analysis of the gut colonization. The relatively small cohort might be a limitation, but in spite of this we demonstrate significant relationships between neonatal colonization by *Bifidobacterium* and increased cytokine responses later in childhood. An additional advantage of our study is that cell stimulations were performed on freshly isolated mononuclear cells to minimize the risk of cell death of certain T cell populations.

In conclusion, our results suggest that early colonization by *Bifidobacterium* may be a marker for a microbial exposure that favors T cell activation in infancy. It remains to be identified what specific factors associated with bacterial gut colonization might prime the T cell population, such as metabolites or bacterial antigens.

## Supplementary Material

Supplemental Material
