# Supplementary material for: Neonatal gut colonization by Bifidobacterium is associated with higher childhood cytokine responses
Source: Gut Microbes. 2020 Dec 4;12(1):1847628. doi: 10.1080/19490976.2020.1847628 (PMC7747801; doi:10.1080/19490976.2020.1847628)
Supplement: Supplemental Material [file KGMI_A_1847628_SM2681.zip › Supplementary information/Rabe et al revised Supplemental Data Tables.docx]

**Supplemental Data**

**Supplemental Table 1. Culture conditions and methods used to identify bacterial species or groups of bacteria**

| **Bacteria** | **Culture conditions** | **Culturing media** | **Microscopical appearance** | **Phenotypic identification** | **Genotypic identification** |
| --- | --- | --- | --- | --- | --- |
| *Staphylococcus aureus* | aerobic  3 days | *Staphylococcus* agar | Gram-positive cocci | positive in coagulase test |  |
| Coagulase-negative Staphylococci | aerobic  3 days | *Staphylococcus* agar | Gram-positive cocci | negative in coagulase test |  |
| *Escherichia coli* | aerobic  3 days | Drigalski agar | Gram-negative rods | API30E |  |
| *Enterobacteraceae*  other than *E. coli* | aerobic  3 days | Drigalski agar | Gram-negative rods | API30E |  |
| *Enterococcus* spp | aerobic  3 days | Enterococcosel  agar | Gram-positive cocci | Hydrolyze esculin on enterococcosel agar |  |
| *Bacteroides* spp | anaerobic  3 days | *Bacteroides* Bile  Esculin agar | Gram-negative rods | RAPID ID32A |  |
| *Bifidobacterium spp* | anaerobic  3 days | Beerens agar | Gram-positive rods |  | *Bifidobacterium*-specific PCR |
| Lactobacillus spp | anaerobic  3 days | Rogosa agar | Gram-positive rods |  | Positive in a *Lactobacillus*-group specific PCR |
| *Clostridium* spp | anaerobic  3 days | alcohol-treated and serially diluted feces cultured anaerobically on Brucella blood agar | Gram-positive or Gram-variables rods | RAPID ID 32A |  |
| *Clostridium difficile* | anaerobic  3 days | CCFA agar | Gram-positive or Gram-variables rods | RAPID ID 32A |  |
| yeast | aerobic  3 days | Saboraud agar  (supplemented with antibiotics) | Yeasts |  |  |

**Supplemental Table 2. Reagents and PCR conditions used in**

**the first and second PCR amplification**

|  | **First PCR** |  | **Second PCR** |
| --- | --- | --- | --- |
| **PCR reagents:** |  |  |  |
| Reaction volume | 20µl |  | 20.4µl |
| 1xKappa HiFi HotStart Readymix | 1 |  | 1 |
| Primers | 5µM (of each primer) |  | 10µM (of each primer) |
| Genomic DNA | 20ng |  | - |
| DNA product form PCR reaction 1 | - |  | 0.2 µl |
|  |  |  |  |
| **PCR conditions:** |  |  |  |
| Denaturation | 3min 95^o^C |  | 3min 95^o^C |
| Denaturation | 25 cycles 30sec 95^o^C |  | 7 cycles 30sec 95^o^C |
| Anneling | 30sec 55^o^C |  | 30sec 55^o^C |
| Elongation | 30sec 72^o^C |  | 30sec 72^o^C |
| Elongation | 5min 72^o^C |  | 5min 72^o^C |

**Supplemental Table 3. Primer design used in the second PCR (outer PCR)**

| **Primer ID** | **Forward primer sequence** |  | **Primer ID** | **Revers primer sequence** |
| --- | --- | --- | --- | --- |
| **1** | AATGATACGGCGACCACCGAGATCTACAC**GGAGTGGGF***TCGTCGGCAGCGTC* |  | **1** | CAAGCAGAAGACGGCATACGAGAT**TGGTGGTG***GTCTCGTGGGCTCGG* |
| **3** | AATGATACGGCGACCACCGAGATCTACAC**AGCGTTTA***TCGTCGGCAGCGTC* |  | **3** | CAAGCAGAAGACGGCATACGAGAT**CCTAAGTA***GTCTCGTGGGCTCGG* |
| **3** | AATGATACGGCGACCACCGAGATCTACAC**TCTACTAA***TCGTCGGCAGCGTC* |  | **3** | CAAGCAGAAGACGGCATACGAGAT**AATAATGA***GTCTCGTGGGCTCGG* |
| **4** | AATGATACGGCGACCACCGAGATCTACAC**TAGTCAGC***TCGTCGGCAGCGTC* |  | **4** | CAAGCAGAAGACGGCATACGAGAT**TCGTTTAT***GTCTCGTGGGCTCGG* |
| **5** | AATGATACGGCGACCACCGAGATCTACAC**CAATTAAA***TCGTCGGCAGCGTC* |  | **5** | CAAGCAGAAGACGGCATACGAGAT**TTCCTGTT***GTCTCGTGGGCTCGG* |
| **6** | AATGATACGGCGACCACCGAGATCTACAC**TATATCAA***TCGTCGGCAGCGTC* |  | **6** | CAAGCAGAAGACGGCATACGAGAT**TAATTTAC***GTCTCGTGGGCTCGG* |
| **7** | AATGATACGGCGACCACCGAGATCTACAC**GCTGAAAT***TCGTCGGCAGCGTC* |  | **7** | CAAGCAGAAGACGGCATACGAGAT**CATGAGAT***GTCTCGTGGGCTCGG* |
| **8** | AATGATACGGCGACCACCGAGATCTACAC**ATGATTAA***TCGTCGGCAGCGTC* |  | **8** | CAAGCAGAAGACGGCATACGAGAT**AAATCTTC***GTCTCGTGGGCTCGG* |
| **9** | AATGATACGGCGACCACCGAGATCTACAC**GTAGCAAT***TCGTCGGCAGCGTC* |  | **9** | CAAGCAGAAGACGGCATACGAGAT**GACTACAT***GTCTCGTGGGCTCGG* |
| **10** | AATGATACGGCGACCACCGAGATCTACAC**AATTCATA***TCGTCGGCAGCGTC* |  | **10** | CAAGCAGAAGACGGCATACGAGAT**TGGACCTT***GTCTCGTGGGCTCGG* |
| **11** | AATGATACGGCGACCACCGAGATCTACAC**GAGCATGC***TCGTCGGCAGCGTC* |  |  |  |
| **13** | AATGATACGGCGACCACCGAGATCTACAC**CAATAGCC***TCGTCGGCAGCGTC* |  |  |  |

**Bold**: index

*Cursive*: Adapter

**Supplemental Table 4. Reads, total number of ASVs and OTUs detected for each child in fecal samples collected at 1 week, 1 month and 6 months of age.**

|  | **1week of age** | | | **1 month of age** | | | **6 months of age** | | |
| --- | --- | --- | --- | --- | --- | --- | --- | --- | --- |
| **Sample ID** | **Reads/sample** | **ASVs (DADA2)/sample** | **97% Vsearch (SILVA db)-OTU/sample** | **Reads/sample** | **ASVs (DADA2)/sample** | **97% Vsearch (SILVA db)-OTU/sample** | **Reads/sample** | **ASVs (DADA2)/sample** | **97% Vsearch (SILVA db)-OTU/sample** |
| BF1 | 142 913 | 95 067 | 33 | 123 053 | 80 246 | 50 | 149 008 | 91 979 | 76 |
| BF2 | 130 069 | 94 952 | 51 | 149 140 | 108 303 | 110 | 151 729 | 97 641 | 137 |
| BF3 | 145 214 | 91 429 | 36 | 131 265 | 91 477 | 38 | 102 745 | 73 995 | 74 |
| BF4 | 138 958 | 84 673 | 68 | 169 290 | 101 444 | 79 | 157 513 | 111 770 | 32 |
| BF5 | 158 530 | 107 468 | 52 | 156 750 | 108 052 | 161 | 178 381 | 122 848 | 130 |
| BF6 | 151 444 | 108 053 | 41 | 144 491 | 97 588 | 69 | 162 300 | 107 852 | 125 |
| BF7 | 169 285 | 111 175 | 33 | 129 816 | 80 247 | 46 | 84 361 | 60 006 | 44 |
| BF8 | 150 272 | 104 007 | 32 | 184 533 | 122 114 | 91 | 189 450 | 125 286 | 77 |
| BF9 | 161 552 | 112 399 | 43 | 155 847 | 104 924 | 76 | 202 739 | 154 366 | 84 |
| BF10 | 143 324 | 105 879 | 61 | 178 737 | 128 720 | 103 | 183 989 | 131 392 | 69 |
| BF11 | 185 491 | 132 389 | 39 | 170 570 | 120 041 | 65 | 84 598 | 60 537 | 76 |
| BF12 |  |  |  | 168 010 | 86 510 | 48 | 173 611 | 88 825 | 82 |
| BF13 | 160 310 | 104 839 | 36 | 213 018 | 147 439 | 104 | 192 625 | 132 173 | 46 |
| BF14 | 123 659 | 84 609 | 27 | 104 315 | 69 088 | 30 | 129 553 | 85 116 | 58 |
| BF15 | 129 133 | 78 610 | 58 | 117 941 | 78 662 | 55 | 128 338 | 84 426 | 84 |
| BF16 | 137 158 | 93 842 | 38 | 136 518 | 83 264 | 48 | 102 510 | 75 392 | 49 |
| BF17 | 130 241 | 80 743 | 43 | 128 696 | 80 112 | 60 | 149 357 | 100 455 | 130 |
| BF18 | 216 718 | 131 074 | 71 | 197 717 | 124 355 | 76 | 212 323 | 142 400 | 157 |
| BF19 | 184 077 | 120 614 | 100 | 218 956 | 162 231 | 97 | 187 535 | 124 416 | 114 |
| BF20 | 194 828 | 135 261 | 38 | 219 075 | 146 115 | 51 | 112 636 | 79 603 | 119 |
| BF21 | 136 458 | 91 587 | 55 | 197 315 | 142 141 | 51 | 205 155 | 140 372 | 123 |
| BF22 | 212 024 | 142 461 | 40 | 165 379 | 109 870 | 52 | 193 657 | 130 260 | 105 |
| BF23 | 195 192 | 130 504 | 45 | 198 208 | 137 066 | 58 | 191 991 | 125 005 | 100 |
| BF24 | 194 177 | 143 674 | 44 | 168 627 | 123 995 | 44 | 105 473 | 76 488 | 66 |
| BF25 | 167 955 | 117 223 | 71 | 217 101 | 142 373 | 97 | 216 601 | 151 040 | 150 |
| BF26 | 193 486 | 131 108 | 42 | 183 784 | 128 530 | 35 | 192 974 | 138 563 | 98 |
| BF27 | 141 956 | 64 219 | 28 | 228 626 | 153 194 | 91 | 184 330 | 123 776 | 130 |
| BF28 | 193 115 | 130 684 | 27 | 190 633 | 127 312 | 41 | 122 403 | 88 880 | 115 |
| BF29 | 201 592 | 141 645 | 35 | 196 868 | 141 467 | 75 | 236 252 | 161 310 | 110 |
| BF30 | 207 855 | 139 794 | 44 | 202 881 | 149 587 | 48 | 247 789 | 169 436 | 131 |
| BF31 | 216 033 | 150 718 | 54 | 224 775 | 159 510 | 53 | 204 999 | 137 158 | 66 |
| BF32 | 216 227 | 142 725 | 50 | 215 310 | 154 345 | 39 | 164 734 | 113 180 | 69 |
| BF33 | 200 050 | 135 900 | 51 | 200 516 | 141 807 | 58 | 205 864 | 141 586 | 74 |
| BF34 | 167 544 | 94 121 | 23 | 187 748 | 109 723 | 65 | 202 639 | 115 632 | 130 |
| BF35 |  |  |  | 192 823 | 91 299 | 79 | 186 320 | 93 108 | 140 |
| BF36 | 168 769 | 93 706 | 30 | 165 555 | 90 522 | 72 | 180 677 | 106 167 | 82 |
| BF37 | 178 941 | 98 364 | 27 | 137 994 | 72 337 | 59 | 93 333 | 51 037 | 44 |
| BF39 |  |  |  | 196 238 | 116 926 | 33 | 184 118 | 104 948 | 53 |
| BF40 | 179 797 | 93 420 | 74 |  |  |  | 193 818 | 103 019 | 127 |
| BF41 | 201 400 | 114 373 | 36 | 160 098 | 82 206 | 48 | 163 976 | 92 437 | 75 |
| BF42 | 229 819 | 143 010 | 42 | 170 848 | 96 779 | 46 | 198 325 | 111 003 | 69 |
| BF43 | 194 833 | 118 444 | 25 | 182 072 | 106 855 | 32 | 200 787 | 100 624 | 83 |
| BF44 | 209 343 | 121 599 | 33 | 165 783 | 81 964 | 38 | 162 302 | 100 236 | 82 |
| BF45 | 183 550 | 96 659 | 62 | 199 893 | 105 321 | 57 | 222 595 | 109 379 | 99 |
| BF46 | 190 519 | 102 827 | 40 | 218 880 | 124 608 | 39 | 208 331 | 108 828 | 52 |
| BF47 | 201 946 | 112 378 | 32 | 210 271 | 110 592 | 36 | 224 434 | 135 643 | 66 |
| BF48 | 193 058 | 111 386 | 48 | 160 381 | 94 694 | 43 | 132 933 | 85 029 | 67 |
| BF49 | 173 598 | 94 605 | 38 | 193 739 | 92 958 | 23 | 182 697 | 94 140 | 96 |
| BF50 | 144 897 | 77 769 | 31 | 135 718 | 77 144 | 22 | 109 988 | 54 420 | 43 |
| BF51 | 144 118 | 72 502 | 37 | 127 226 | 66 146 | 43 | 120 431 | 51 489 | 135 |
| BF52 | 170 687 | 88 047 | 33 | 135 793 | 57 816 | 53 | 121 384 | 66 068 | 84 |
| BF53 | 161 506 | 87 305 | 28 | 147 278 | 79 823 | 36 | 162 178 | 85 455 | 44 |
| BF54 | 219 592 | 118 271 | 75 | 208 737 | 115 084 | 43 | 235 171 | 130 911 | 176 |
| BF55 | 171 819 | 74 106 | 28 | 211 990 | 116 939 | 58 | 210 931 | 119 039 | 123 |
| BF56 | 209 053 | 123 115 | 22 | 204 908 | 119 359 | 40 | 117 618 | 67 330 | 56 |
| BF57 | 207 735 | 123 245 | 38 | 206 189 | 112 840 | 53 | 190 545 | 97 876 | 62 |
| BF58 | 202 282 | 107 823 | 36 | 139 280 | 76 677 | 32 | 250 926 | 144 858 | 123 |
| BF59 | 215 603 | 119 148 | 52 | 197 395 | 124 799 | 33 | 210 578 | 115 091 | 77 |
| BF60 | 215 704 | 110 809 | 44 | 204 538 | 107 327 | 30 | 168 329 | 96 212 | 72 |
| BF61 | 198 127 | 109 212 | 42 | 236 011 | 125 366 | 49 | 220 555 | 124 591 | 58 |
| BF62 | 202 780 | 105 782 | 46 | 212 136 | 110 592 | 47 | 220 439 | 104 274 | 59 |
| BF63 | 159 091 | 37 248 | 18 | 249 517 | 146 702 | 62 | 254 210 | 144 511 | 75 |
| BF64 | 222 573 | 103 788 | 40 | 229 175 | 118 736 | 50 | 108 590 | 63 381 | 38 |
| BF65 |  |  |  | 164 184 | 90 254 | 33 | 199 313 | 101 339 | 53 |
| BF66 | 197 767 | 121 275 | 49 | 215 116 | 121 758 | 50 | 243 323 | 139 770 | 67 |
