# Supplementary material for: Neonatal gut colonization by Bifidobacterium is associated with higher childhood cytokine responses
Source: Gut Microbes. 2020 Dec 4;12(1):1847628. doi: 10.1080/19490976.2020.1847628 (PMC7747801; doi:10.1080/19490976.2020.1847628)
Supplement: Supplemental Material [file KGMI_A_1847628_SM2681.zip › Supplementary information/Rabe et al Supplemenal figures second revision.pptx]

## Slide 1
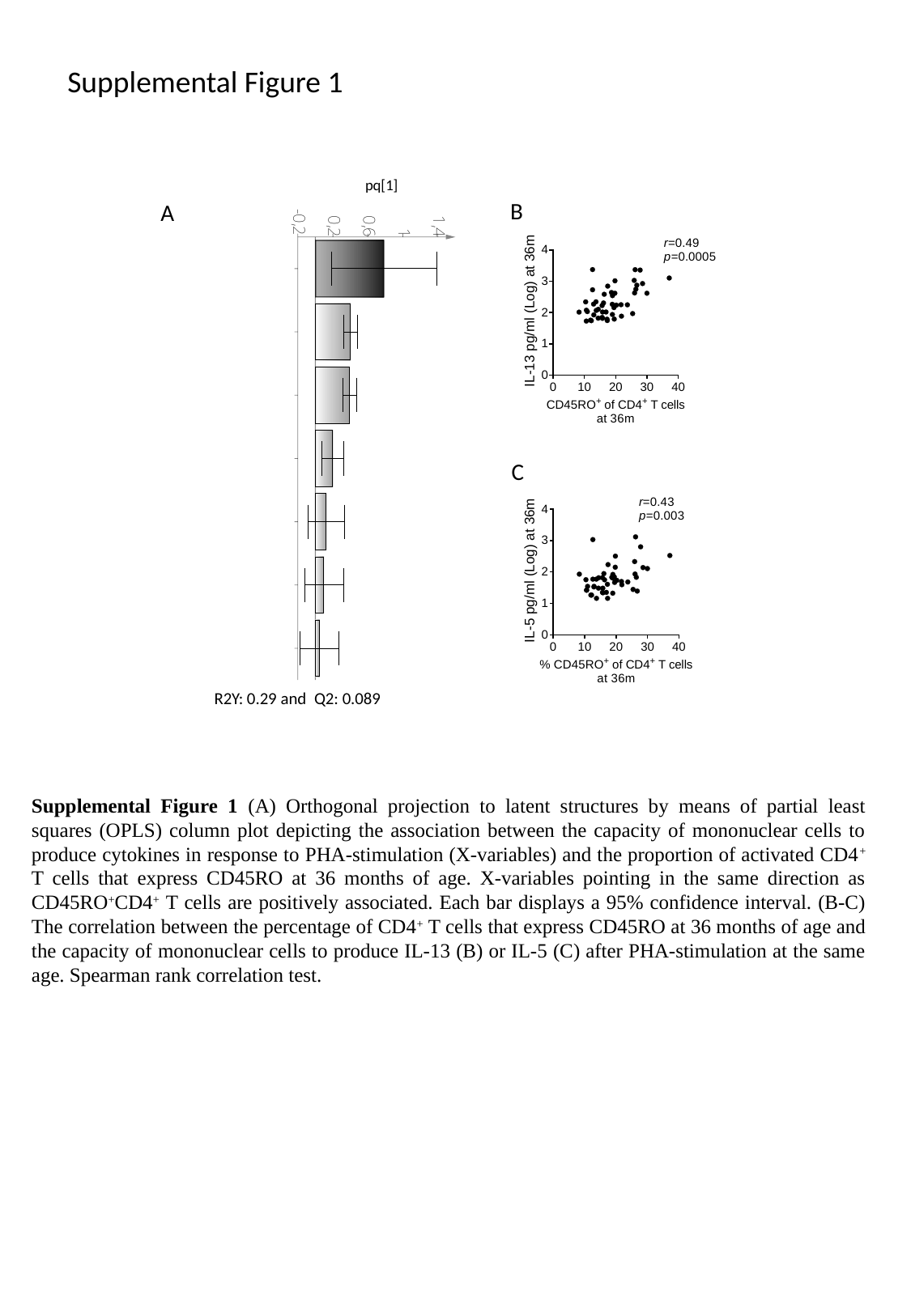

Supplemental Figure 1
pq[1]
B
A
C
R2Y: 0.29 and Q2: 0.089
Supplemental Figure 1 (A) Orthogonal projection to latent structures by means of partial least squares (OPLS) column plot depicting the association between the capacity of mononuclear cells to produce cytokines in response to PHA-stimulation (X-variables) and the proportion of activated CD4+ T cells that express CD45RO at 36 months of age. X-variables pointing in the same direction as CD45RO+CD4+ T cells are positively associated. Each bar displays a 95% confidence interval. (B-C) The correlation between the percentage of CD4+ T cells that express CD45RO at 36 months of age and the capacity of mononuclear cells to produce IL-13 (B) or IL-5 (C) after PHA-stimulation at the same age. Spearman rank correlation test.

## Slide 2
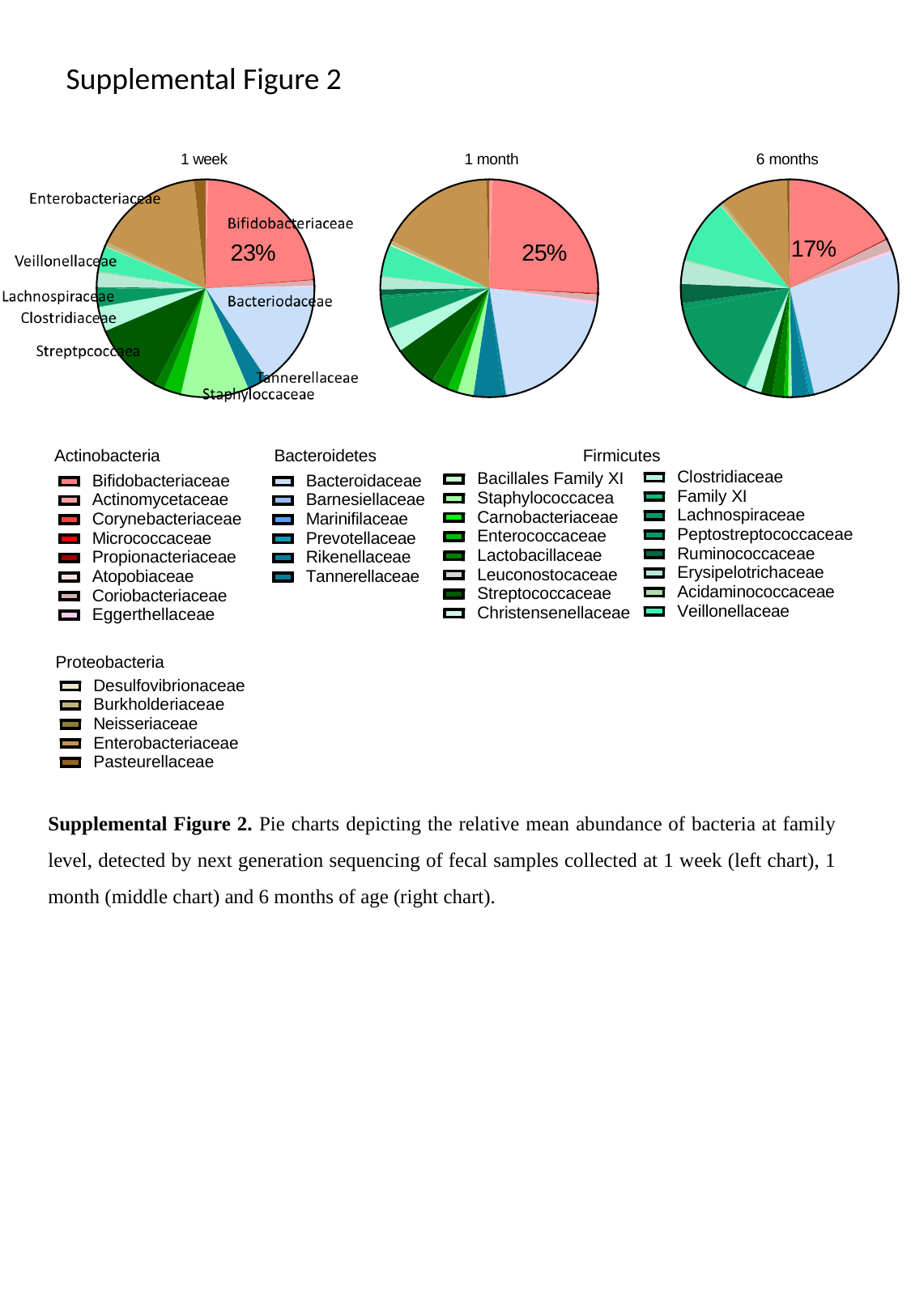

Supplemental Figure 2
Supplemental Figure 2. Pie charts depicting the relative mean abundance of bacteria at family level, detected by next generation sequencing of fecal samples collected at 1 week (left chart), 1 month (middle chart) and 6 months of age (right chart).

## Slide 3
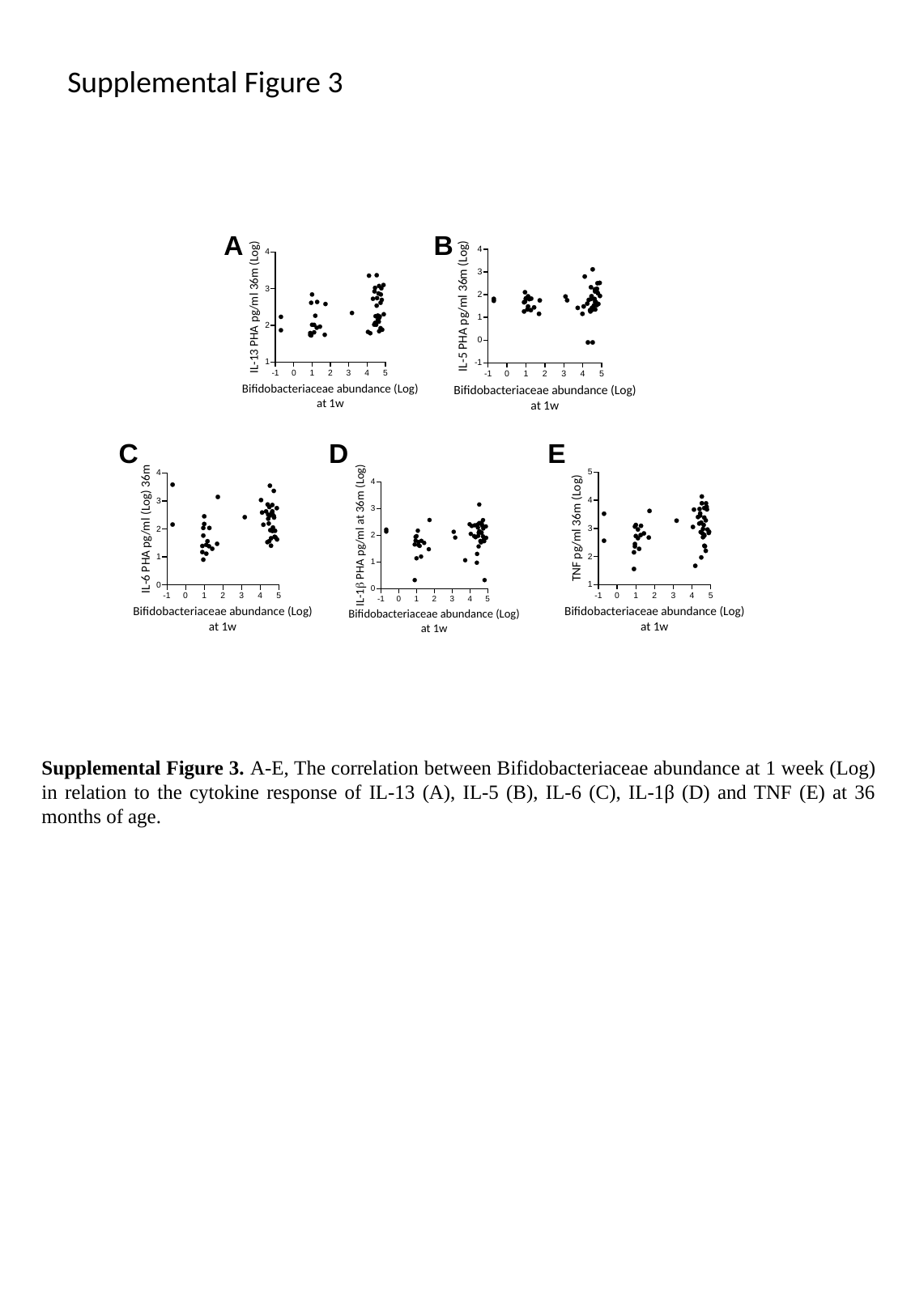

Supplemental Figure 3
Supplemental Figure 3. A-E, The correlation between Bifidobacteriaceae abundance at 1 week (Log) in relation to the cytokine response of IL-13 (A), IL-5 (B), IL-6 (C), IL-1β (D) and TNF (E) at 36 months of age.

## Slide 4
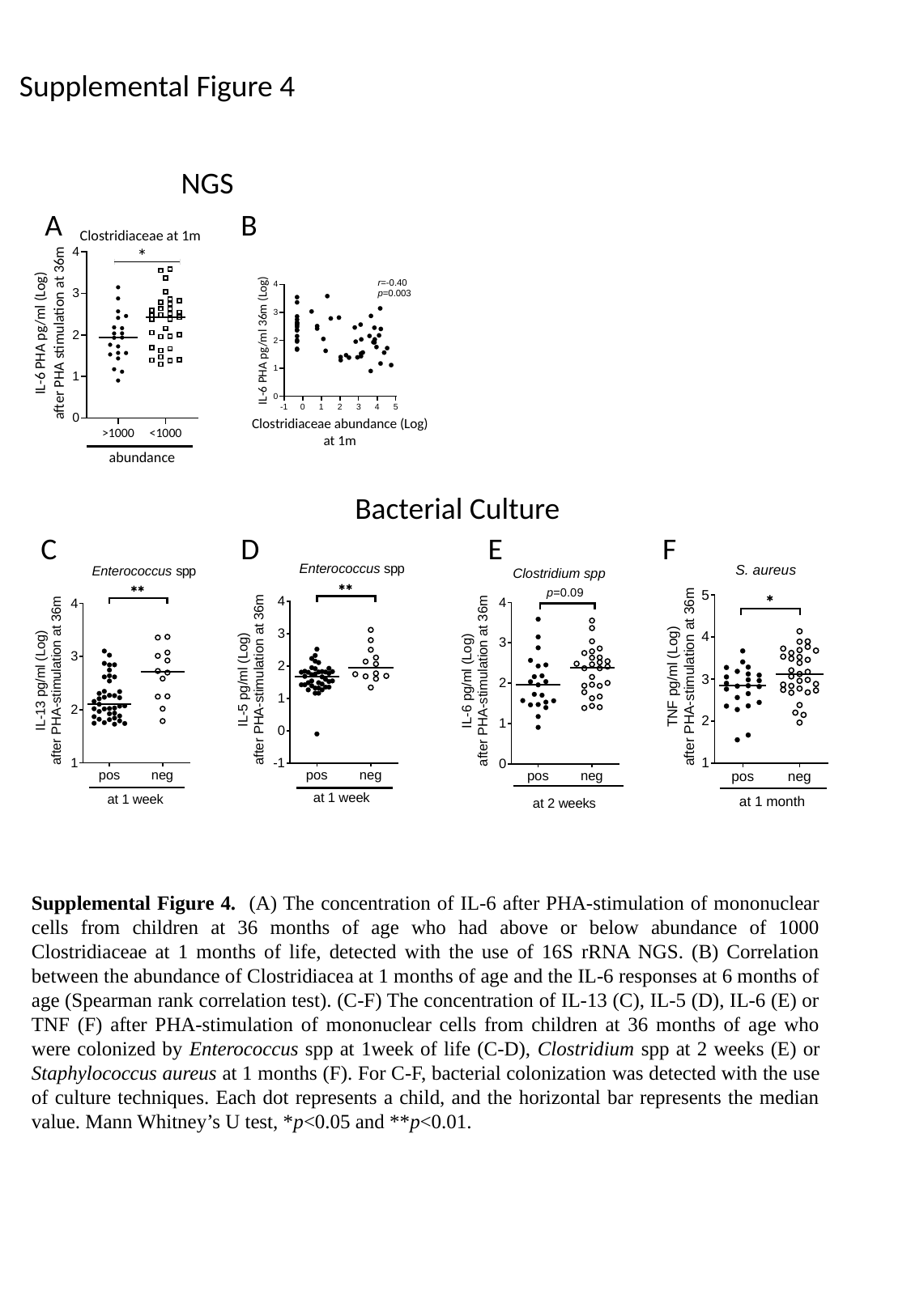

Supplemental Figure 4
NGS
A
B
Bacterial Culture
C
D
E
F
Supplemental Figure 4. (A) The concentration of IL-6 after PHA-stimulation of mononuclear cells from children at 36 months of age who had above or below abundance of 1000 Clostridiaceae at 1 months of life, detected with the use of 16S rRNA NGS. (B) Correlation between the abundance of Clostridiacea at 1 months of age and the IL-6 responses at 6 months of age (Spearman rank correlation test). (C-F) The concentration of IL-13 (C), IL-5 (D), IL-6 (E) or TNF (F) after PHA-stimulation of mononuclear cells from children at 36 months of age who were colonized by Enterococcus spp at 1week of life (C-D), Clostridium spp at 2 weeks (E) or Staphylococcus aureus at 1 months (F). For C-F, bacterial colonization was detected with the use of culture techniques. Each dot represents a child, and the horizontal bar represents the median value. Mann Whitney’s U test, *p<0.05 and **p<0.01.

## Slide 5
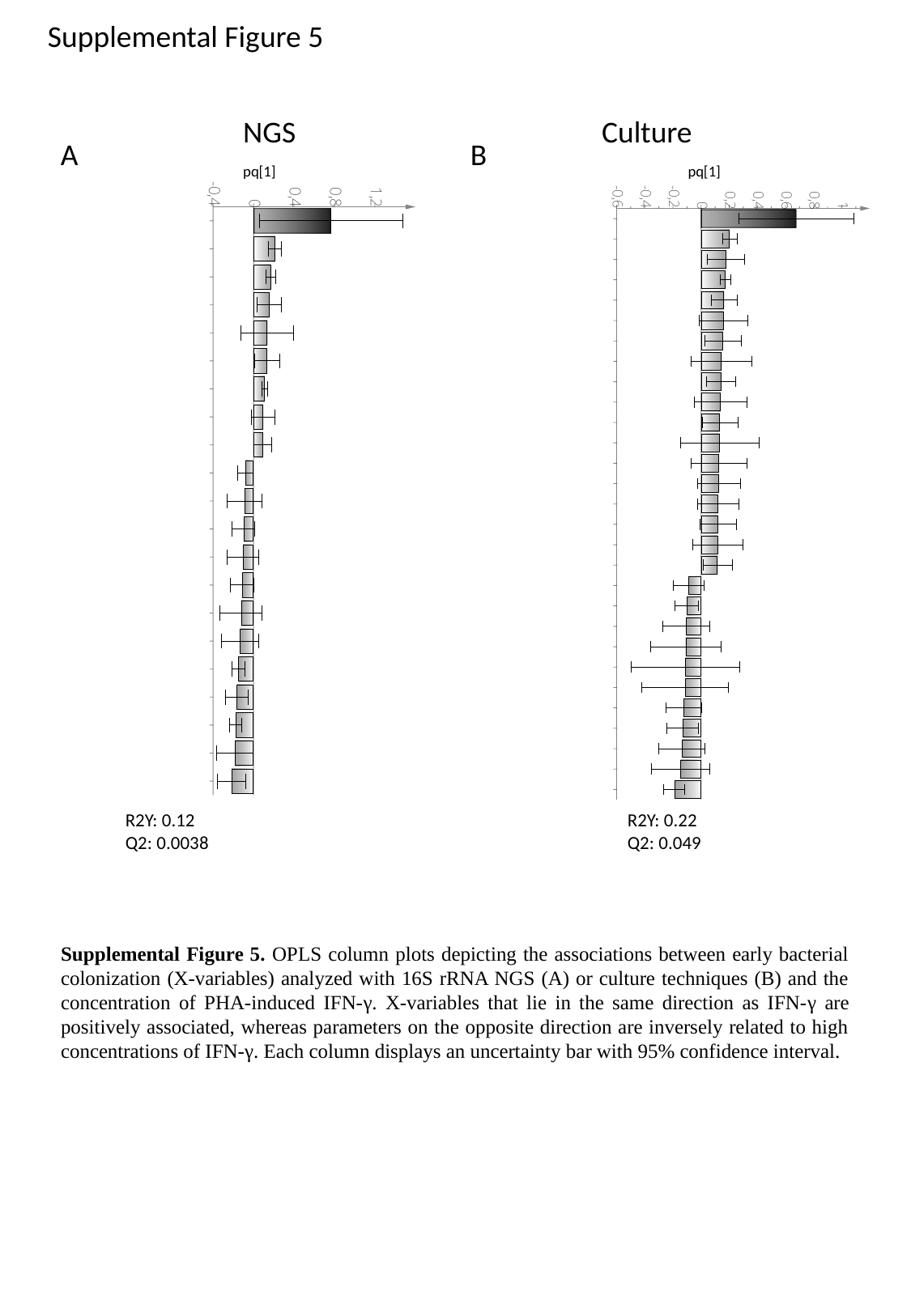

Supplemental Figure 5
NGS
Culture
A
B
pq[1]
pq[1]
R2Y: 0.12
Q2: 0.0038
R2Y: 0.22
Q2: 0.049
Supplemental Figure 5. OPLS column plots depicting the associations between early bacterial colonization (X-variables) analyzed with 16S rRNA NGS (A) or culture techniques (B) and the concentration of PHA-induced IFN-γ. X-variables that lie in the same direction as IFN-γ are positively associated, whereas parameters on the opposite direction are inversely related to high concentrations of IFN-γ. Each column displays an uncertainty bar with 95% confidence interval.

## Slide 6
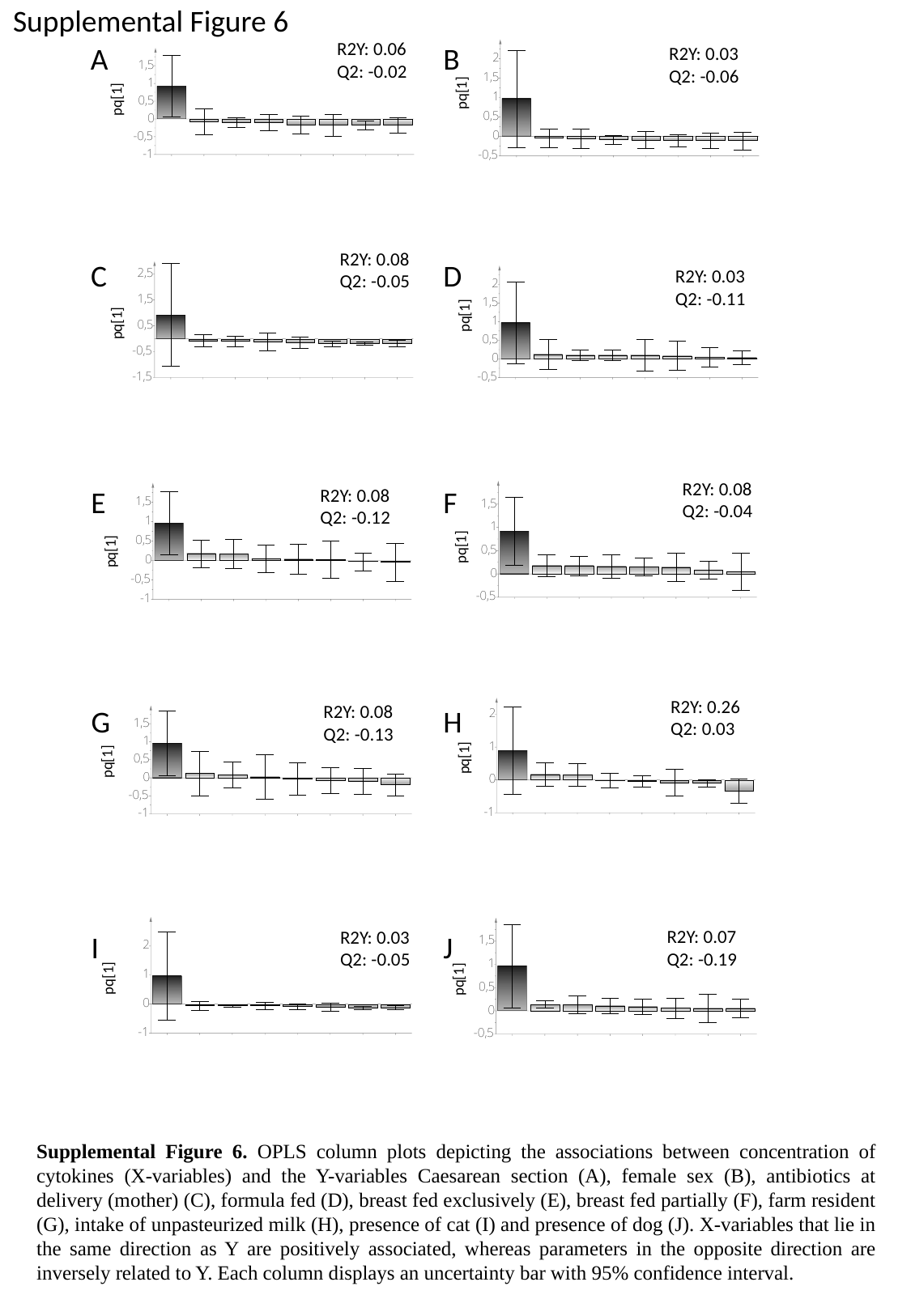

Supplemental Figure 6
R2Y: 0.06
Q2: -0.02
pq[1]
A
B
R2Y: 0.03
Q2: -0.06
pq[1]
R2Y: 0.08
Q2: -0.05
pq[1]
C
D
R2Y: 0.03
Q2: -0.11
pq[1]
R2Y: 0.08
Q2: -0.04
pq[1]
E
F
R2Y: 0.08
Q2: -0.12
pq[1]
R2Y: 0.26
Q2: 0.03
pq[1]
R2Y: 0.08
Q2: -0.13
pq[1]
G
H
R2Y: 0.03
Q2: -0.05
pq[1]
R2Y: 0.07
Q2: -0.19
pq[1]
I
J
Supplemental Figure 6. OPLS column plots depicting the associations between concentration of cytokines (X-variables) and the Y-variables Caesarean section (A), female sex (B), antibiotics at delivery (mother) (C), formula fed (D), breast fed exclusively (E), breast fed partially (F), farm resident (G), intake of unpasteurized milk (H), presence of cat (I) and presence of dog (J). X-variables that lie in the same direction as Y are positively associated, whereas parameters in the opposite direction are inversely related to Y. Each column displays an uncertainty bar with 95% confidence interval.
